# Supplementary material for: Correction: Beyond Muscles Stiffness: Importance of State-Estimation to Account for Very Fast Motor Corrections
Source: PLoS Comput Biol. 2014 Oct 30;10(10):e1003996. doi: 10.1371/journal.pcbi.1003996 (PMC4214588; doi:10.1371/journal.pcbi.1003996)
Supplement: File S2 — Republished corrected article. (PDF) [file pcbi.1003996.s002.pdf]

OPEN ACCESS Freely available online

PLOS | COMPUTATIONAL BIOLOGY

Beyond Muscles Stiffness: Importance of State-Estimation to Account for Very Fast Motor Corrections

Frédéric Crevecoeur[1], Stephen H. Scott[1,2]*

1 Centre for Neuroscience Studies, Queen's University, Kingston, Canada, **2** Department of Biomedical and Molecular Sciences, Queen's University, Kingston, Canada

Abstract

Feedback delays are a major challenge for any controlled process, and yet we are able to easily control limb movements with speed and grace. A popular hypothesis suggests that the brain largely mitigates the impact of feedback delays (~50 ms) by regulating the limb intrinsic visco-elastic properties (or impedance) with muscle co-contraction, which generates forces proportional to changes in joint angle and velocity with zero delay. Although attractive, this hypothesis is often based on estimates of limb impedance that include neural feedback, and therefore describe the entire motor system. In addition, this approach does not systematically take into account that muscles exhibit high intrinsic impedance only for small perturbations (short-range impedance). As a consequence, it remains unclear how the nervous system handles large perturbations, as well as disturbances encountered during movement when short-range impedance cannot contribute. We address this issue by comparing feedback responses to load pulses applied to the elbow of human subjects with theoretical simulations. After validating the model parameters, we show that the ability of humans to generate fast and accurate corrective movements is compatible with a control strategy based on state estimation. We also highlight the merits of delay-uncompensated robust control, which can mitigate the impact of internal model errors, but at the cost of slowing feedback corrections. We speculate that the puzzling observation of presynaptic inhibition of peripheral afferents in the spinal cord at movement onset helps to counter the destabilizing transition from high muscle impedance during posture to low muscle impedance during movement.

Citation: Crevecoeur F, Scott SH (2014) Beyond Muscles Stiffness: Importance of State-Estimation to Account for Very Fast Motor Corrections. PLoS Comput Biol 10(10): e1003869. doi:10.1371/journal.pcbi.1003869

Editor: Francisco J. Valero-Cuevas, University of Southern California, United States of America

Received April 9, 2014; **Accepted** August 20, 2014; **Published** October 9, 2014

Copyright: © 2014 Crevecoeur, Scott. This is an open-access article distributed under the terms of the Creative Commons Attribution License, which permits unrestricted use, distribution, and reproduction in any medium, provided the original author and source are credited.

Data Availability: The authors confirm that all data underlying the findings are fully available without restriction. The experimental data and the table of muscle parameters can be downloaded from the following public folder: http://limb.biomed.queensu.ca/publications/data_fc2014/.

Funding: FC receives a salary fellowship award from the Canadian Institute of Health Research (http://www.cihr-irsc.gc.ca/, fellowship code: 201102MFE-246249-210313). SHS holds a GlaxoSmithKlein Chair in Neuroscience. The funders had no role in study design, data collection and analysis, decision to publish, or preparation of the manuscript.

Competing Interests: SHS is associated with BKIN Technologies that commercializes the robotic equipment used for the experiments.

* Email: steve.scott@queensu.ca

Introduction

The presence of sensory and motor delays in any control process can lead to highly unstable behavior [1]. Impressively, humans (and other animals) are able to make rapid corrective responses even with sensorimotor delays on the order of 50 ms [2,3]. Several hypotheses have been formulated, each making distinct predictions about how the nervous system handles sensorimotor delays. One common view argues that the brain exploits the spring-like properties of muscle to stabilize the body during motor control, commonly termed impedance control [4]. In this framework, the brain controls the state of the peripheral motor apparatus in such a way that the intrinsic biomechanical properties of the limb restore a force proportional to changes in joint angle (stiffness) and velocity (viscosity) with zero delay [4–9].

In order to avoid ambiguous terminology, we will use *impedance* to refer to muscle's intrinsic visco-elastic properties, therefore excluding motor responses mediated by neural feedback [4,10,11]. It is important to stress that there is some confusion in the literature relative to the definition of impedance control. Many studies include not only the stiffness related to muscle activation, but implicitly also neural feedback as a factor contributing to limb impedance [5,7,8,12–18]. This is because these studies use estimates of joint stiffness and viscosity based on perturbation responses that last >200 ms [12], and thus depend on neural feedback including the short-latency (~20 ms–50 ms), long-latency (~50 ms–100 ms) and early voluntary responses (> 100 ms). This methodology is now questionable given recent observations on the sophistication of long-latency and early voluntary responses [2]. Also, long-latency responses are known to involve cortical and cerebellar circuits involved in voluntary control [19,20]. Thus, estimates of limb impedance based on motor responses beyond ~50 ms include essentially the entire motor system, peripheral and central.

Using our definition of muscle impedance, it is clear that the conventional perturbation technique does not provide estimates of the intrinsic muscles properties. Thus it is important to re-evaluate the contribution of muscles' intrinsic impedance independent of neural feedback in order to better understand how the nervous system counters perturbations during motor control.

A challenge in modeling the stiffness properties of muscle is that their properties vary with changes in muscle length: in vivo studies highlight relatively high stiffness for small perturbations corresponding to less than only a few degrees of joint motion (short-range stiffness, [21,22]), whereas larger perturbations must rely on relatively low stiffness properties associated with the muscle's

Author Summary

Recent studies have investigated how the brain generates purposeful feedback responses to perturbations during motor control. One hypothesis suggests that the brain exploits the spring-like properties of muscles to counter perturbations. However, muscles exhibit high mechanical impedance only against small perturbations during posture, which questions the general contribution of intrinsic muscle impedance for feedback control. Alternatively, the brain may directly map sensory data into motor commands without compensating for sensorimotor delays, which is known to limit control performance. A third hypothesis suggests that neural activity following an external disturbance estimates the current state of the limb to generate a motor response. We used a perturbation paradigm where healthy participants were instructed to respond to perturbations within an extremely short time window. Comparing participants' performances with a model considering intrinsic joint impedance and conduction delays revealed that the case of state estimation was the best candidate control model to explain very fast corrective response of humans. This study emphasizes that model-based control can generate human-like rapid and stable feedback responses given low muscle stiffness and sensorimotor delays.

force-length/velocity curves [21–24]. Taking these limitations into account, it remains unclear how the brain generates fast and stable feedback responses to external disturbances, in particular when perturbations exceed the short-range impedance.

To address this issue, we first illustrate how changes in muscle impedance dramatically alter the capabilities of muscles' intrinsic properties to oppose external disturbances, such that stable corrections for small disturbances abruptly switch to slow and oscillatory responses following the transition from high to low impedance that occurs beyond the short-range. Next, we characterize the performance of healthy humans instructed to counter moderate-sized perturbations, highlighting the ability of humans to make very rapid and stable motor corrections. Finally, we investigate whether different feedback control mechanisms can generate human-like corrective responses, considering long-latency delays (~50 ms) and intrinsic joint impedance observed beyond the short-range stiffness. We show that the model including a state estimator was the best candidate to reproduce fast motor responses of humans following abrupt perturbations inducing large motor errors. Essentially, participants were able to increase their feedback gains without altering the kinematics of corrective movements, which we show is the signature of state estimators. We also suggest that impedance control of muscle can be beneficial during postural control against small perturbations. Beyond the short-range stiffness, our data and simulations suggest that fast and stable feedback control requires internal models and state estimation to compensate for low impedance and sensorimotor delays.

Results

Transition to Low Stiffness Impacts Limb Trajectory

Muscles perturbed in vivo display high-impedance over a short range, a property commonly referred to as short-range stiffness [21,22,25]. Beyond this short range, the intrinsic impedance of muscle drops dramatically, and depends on its force-length and force-velocity properties [23,24]. Data from the cat soleus muscle suggest that the short-range impedance corresponds to ~1 mm of muscle stretch (Figure 1A, schematic redrawn of Figure 2 from [22]), which corresponds to ~2.6% of its fascicles length [23]. Transposed to human elbow muscles (see Methods), these numbers suggest that the elbow joint exhibits high intrinsic impedance when changes in angle are less than 5 deg in amplitude (see also [26]).

The transition from high to low impedance has a direct impact on corrective trajectories generated by intrinsic muscle properties [4]. Indeed, transient perturbations inducing changes in joint angles >5 degrees dramatically reduce the potential contribution of muscle intrinsic impedance to the corrective response. Figure 1 B displays the simulated perturbation-related changes in joint angle following application of small- (gray) and medium-sized (black) perturbations. For these simulations, we considered both elastic and viscous terms to describe the short-range intrinsic properties, and therefore refer to it as short-range *impedance* (see also Methods). The values of the exemplar perturbations in Figure 1 B were chosen so that the motion either maintained muscles within its short range (high impedance), or exceeded 5 degrees, transitioning muscle to low impedance. Observe the important difference in joint trajectories induced by the change in muscle visco-elastic properties. The peak-to-peak change in joint angle and time to first zero-crossing are markedly altered following the transition from high to low impedance. These two variables are plotted as a function of pulse magnitude in Figure 1C to further illustrate the bifurcation in kinematics parameters resulting from the transition from high to low muscle impedance. This emergent consequence of changes in muscle intrinsic properties on joint motion clearly emphasizes the need for central compensation for biomechanical features of the motor system.

Thus, impedance control may provide stability when perturbations induce small amounts of joint motion. Against larger disturbances, low muscle impedance would generate slow and oscillatory corrections clearly incompatible with human motor behaviour. The following sections present human motor responses to perturbations and address how the nervous system may handle the low muscle impedance along with the additional problem related to temporal delays in sensorimotor transmission.

Human Experiment

Main experiment. The purpose of the human experiment was to quantify the ability of humans to generate rapid corrections against external perturbations in order to compare their performance to various control strategies. Participants interacted with a robotic exoskeleton supporting their arm against gravity and allowing motion in the horizontal plane (Figure 2 A). Visual targets and a hand-aligned cursor were projected on a virtual reality display aligned with the workspace of the arm. The shoulder joint was physically locked and perturbation pulses (Figure 2 B) were applied to the elbow joint. Perturbations were applied with three different background loads used to pre-excite specific muscle groups (+2 Nm, 0 Nm −neutral condition – and − 2 Nm). Participants were instructed to stabilize their fingertips in the start target and return to the goal target following the perturbations (Figure 2 C) within a moderate (600 ms) or very short (300 ms) amount of time. The latter time constraint was used to induce an increase in feedback gains [27], and compare the resulting movement profiles with theoretical simulations. Details about the experimental procedures are provided in the Methods section.

Average traces of the elbow motion are represented in Figure 3 A and B. The 600 ms condition was easy and participants obtained 95% successful trials on average (range: 85–100%). Maximum elbow displacement was 14.5±3.7 deg degrees (mean ± SD, range 8–20.4, Figure 3D) and return time was 400±43 ms.

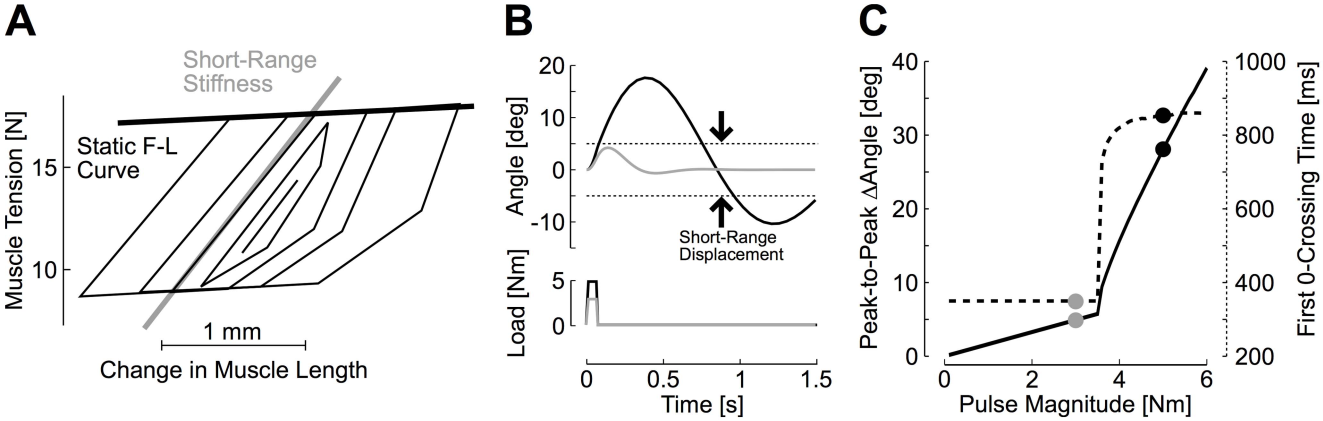

Figure 1. Short-range stiffness. A: Relationship between muscle force and changes in muscle length for different perturbation amplitudes. Traces represent the changes in tension following cyclical changes in fascicle length of varying amplitude. Data were schematically reproduced from Rack and Westbury [12]. The short-range stiffness (gray) and static force-length curve (black) are represented. B: Perturbation-related motion following perturbation pulses applied on a simulated single joint system with high stiffness in the short range (<5 deg), and low stiffness beyond the short range. The gray (3 Nm) and black (5 Nm) perturbation pulses were chosen to illustrate the impact of rapid changes in muscles properties. See Methods for details about the derivation from muscles parameters (fascicle length, physiological cross sectional area and moment arm). C: Peak-to-peak joint displacement (solid, left axis) and first zero-crossing time (dashed, right axis) as a function of the perturbation magnitude under the hypothesis of limb-impedance control. The parameters corresponding to single trajectories plotted in Panel B are reported with similar color code (gray: 3 Nm, black: 5 Nm).
doi:10.1371/journal.pcbi.1003869.g001

In contrast, the 300 ms condition was challenging and the success rate dropped to 49±25% (mean ± SD across participants). Maximum elbow displacement was reduced in this condition (11.6±3.2 degrees, range 7.3–17.4, Figure 3D). The median return time (i.e. the elapsed time outside of the target) was 270 ms±11 ms (mean ± SD across subjects Figure 2 C). The increase in feedback gains associated with this condition generated a decrease in maximum joint angle for 12/15 participants

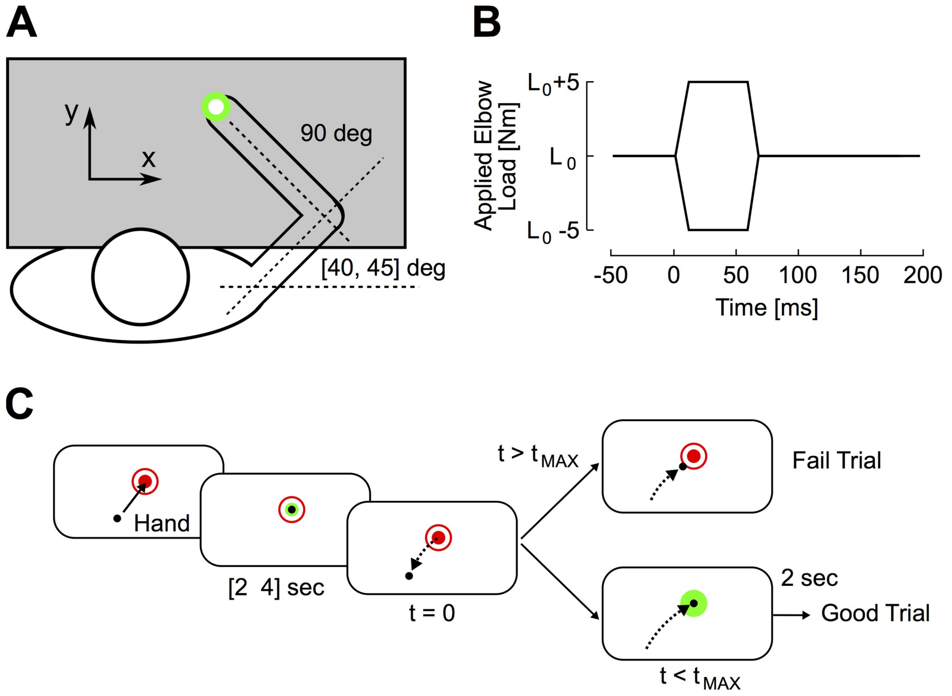

Figure 2. Experimental procedure. A: Overhead representation of a participant performing the task. The hand aligned cursor (white dot) and the visual targets were projected on the virtual reality display and direct vision of the arm was blocked. B: Perturbation pulses applied on the elbow joint (±5 Nm, 50 ms with linear ramp-up/down of 10 ms) were added to the background torque (L0 = +2, 0 or −2 Nm, see Methods). C: Illustration of the time course of a trial. Participants were instructed to reach for the centre of the start target (red dot). The perturbation was applied after a random time delay ranging from 2 sec to 4 sec. Participants had to return to the goal target (red circle) within the prescribed time constraint (t_{MAX} = 300 ms or 600 ms), and stabilize in this target for 2 sec. A green or red goal target indicated success or failure to meet the timing criterion, respectively.
doi:10.1371/journal.pcbi.1003869.g002

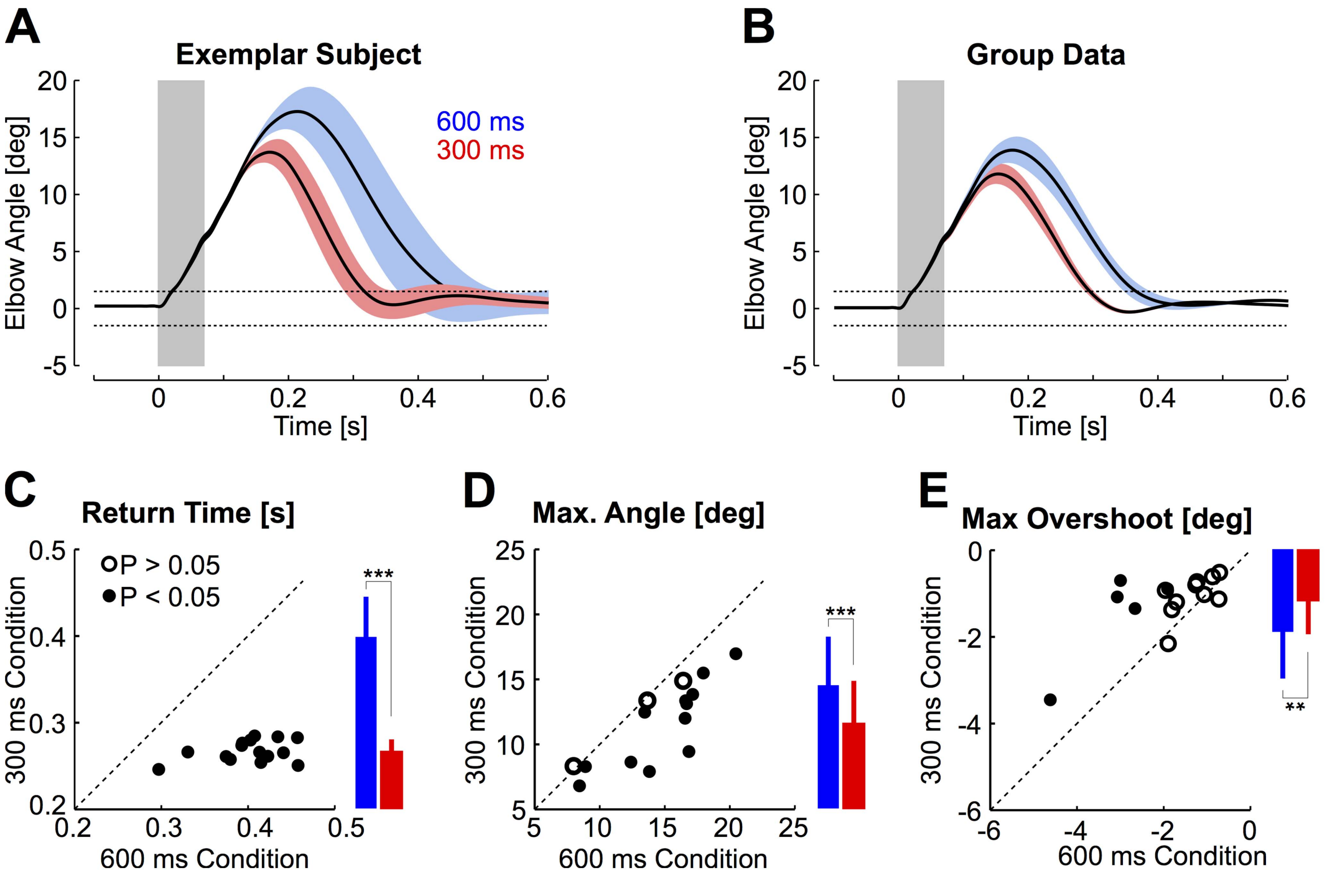

Figure 3. Movement kinematics. A: Average elbow motion from one representative subject. The shaded areas represent 1 standard deviation across trials (blue: 600 ms condition, red: 300 ms condition). The gray rectangle illustrates the duration of the perturbation pulse (70 ms, see Figure 1B). The dashed horizontal lines represent the 3 deg window used to determine the return times and validate the trials offline (see Methods). B: Same as A for group subjects' data. Shaded areas represent 1 standard error across subjects. Panels A and B included all trials. The effect of perturbation offset can be observed as small deviations at the end of the pulse duration (gray rectangle). C: Return times from the 300 ms condition plotted against the return times from the 600 ms condition. Filled dots indicate significant effect across conditions from individual trials (Wilcoxon ranksum test, $P<0.05$). Data summary in the form of bar plots are presented using the same vertical axis as the scatter plot. Color codes correspond to Panels A and B, and stars indicate significant difference across conditions (3 starts: $P<0.001$, 2 stars: $P<0.01$). Vertical bars represent one standard deviation across participants. D: Same as C for the maximum elbow displacement. E: Same as C for the maximum target overshoot. Only successful trials were included in panels C to E.
doi:10.1371/journal.pcbi.1003869.g003

(Figure 3 D), followed by similar or reduced absolute target overshoot across conditions (Figure 3 E). Group comparisons confirmed a clear reduction in return times relative to the 600 ms condition (paired t-test, $t_{(14)}=-12$, $P<0.001$), followed by a reduction in maximum joint angle ($t_{(14)}=-5.1$, $P<0.001$) and a reduction in absolute target overshoot ($t_{(14)}=3.49$, $P<0.01$). Thus, participants generated faster corrective movements without substantially altering the shape of the corrective movement. Similar conclusions characterize participants' behaviour after including the non-successful trials in the dataset. We observed a significant reduction in return times ($t_{(14)}=-8$, $P<0.001$) and a significant reduction in maximum elbow angle across conditions of temporal constraints ($t_{(14)}=-4.6$, $P<0.001$), whereas the target overshoot was statistically similar across conditions ($t_{(14)}=1.22$, $P=0.24$).

The control of limb intrinsic impedance is often assumed to depend on co-activation of antagonist muscles groups [10–12]. We recorded the activity of the main muscles spanning the elbow joint to quantify how much participants spontaneously relied on this strategy. We observed a significant increase in baseline activity for 7/15 participants between the 600 ms and 300 ms conditions (Wilcoxon ranksum test on average baseline activity across trials, Figure 4). Looking at the average across the four muscles, we found that 3/15 participants spontaneously increased their baseline activity by more than 50% of the activity recorded in the 600 ms condition. The same analysis performed on individual muscle samples revealed similar results, with 15/60 individual samples displaying >50% increase in baseline activity. Group data confirmed a significant increase in baseline muscle activity corresponding to 0.14 ± 0.21 a.u. (mean ± SD, Figure 4, paired t-test on individual means, $t_{(14)}=2.5$, $P<0.05$). However, it is important to note that this increase is quite small. Indeed, an average of 14% of the activity evoked by a 2 Nm constant load corresponds to a change in force required to compensate for the weight of a 70 g mass placed 40 cm away from the elbow joint, which corresponds to holding a small object such as a plum in one's hand (assuming no background noise in the EMG signals). Notably, the spontaneous increase in co-contraction did not correlate with success rate in the 300 ms condition (linear regression on participants' individual means, $P=0.58$), and the return times were negatively correlated with the baseline activity for 7/15 subjects (linear regressions on individual trials from the same condition, $P<0.05$). Thus the link between muscle

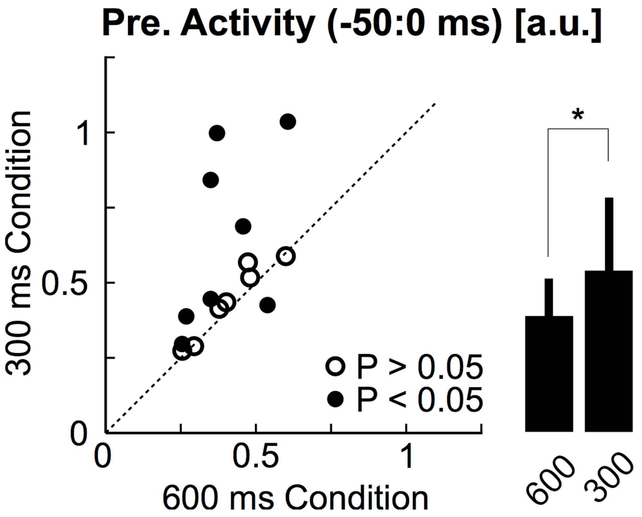

Figure 4. Effect of timing condition on muscle co-activation. Average pre-perturbation activity (from −50 ms to 0 ms) in the 300 ms condition plotted against the pre-perturbation activity in the 600 ms condition. Filled dots designate participants for whom the change in pre-perturbation activity was significant (Wilcoxon ranksum tests on individual trials, $P<0.05$). Open dots represent participants who did not display any significant change in pre-perturbation activity. The bar plot summarizes the effect of the timing condition on the pre-perturbation activity (mean ± SD across participants). The star indicate significant increase (one-tail paired t-test, $P<0.05$).
doi:10.1371/journal.pcbi.1003869.g004

co-activation and behavioural performance, often assumed under the hypothesis of impedance control, was not a strong feature of our dataset.

Although the average spontaneous increase in baseline activity was small, some participants clearly used this strategy and increased their baseline to ~1 a.u., corresponding to the activity evoked by a 2 Nm background load (Figure 4). We investigated further possible effects of pre-activation on the movement kinematics by applying a constant background load of 2 Nm on the elbow (Figure 2 B, $L_0 = \pm 2$ Nm, see Methods). Average joint motion and muscle responses are shown in Figure 5 A and 5 B for the 300 ms condition. The muscle pre-perturbation activity was 0.53±0.23 a.u. greater with the application of a background load (mean ± SD across participants, Figure 5 B, inset, $t_{(14)} = 8.8$, $P<0.001$). Pre-loading the muscles significantly decreased the maximum change in elbow angle ($t_{(14)} = -5.73$, $P<0.001$), but also induced an absolute increase in target overshoot ($t_{(14)} = -2.4$, $P<0.05$). As a result, the return times ($t_{(14)} = 1$, $P = 0.32$) and the success rate ($t_{(14)} = 1.26$, $P = 0.23$) did not display any statistical change across pre-loading conditions. This analysis indicates that the spontaneous increase in pre-perturbation activity reported in Figure 4 (~0.14 a.u. on average) likely played a minor role in the behavioural performance.

Control experiment. Applying a background load on the participants' forearm in the main experiment allowed us to address whether increases in baseline activity impacted motor corrections while controlling accurately the pre-perturbation activity. However, it is possible that motor responses in this case differ from those following muscles co-activated in the absence of a background load. To address the influence of co-contraction more directly, we instructed participants to perform a block of trials while co-activating elbow muscles. This instruction induced important changes in pre-perturbation activity despite large variability across participants. The substantial increase in baseline activity also induced a clear increase in the short-latency stretch reflex across conditions. This comparison was made with the response collected in the condition when the antagonist muscle group was pre-loaded to magnify the difference resulting from changes in muscle state (Figure 6A). We refer to this pre-perturbation muscle state as the unloaded condition. Surprisingly, elbow motion was minimally affected until ~85 ms (Figure 6B and C, black arrow). The relationship between changes in baseline activity and changes in joint angle at 130 ms confirms that co-activation is beneficial to limit perturbation-related motion (Figure 6C), but likely through recruitment of short-latency feedback.

We found that onset of divergence between elbow motion across conditions occurred before 20 ms for 2/6 participants shown in green in Figure 6C (ROC on individual trials, see Methods), which is sufficiently fast to be attributed to changes in intrinsic properties. The same two participants were able to limit the

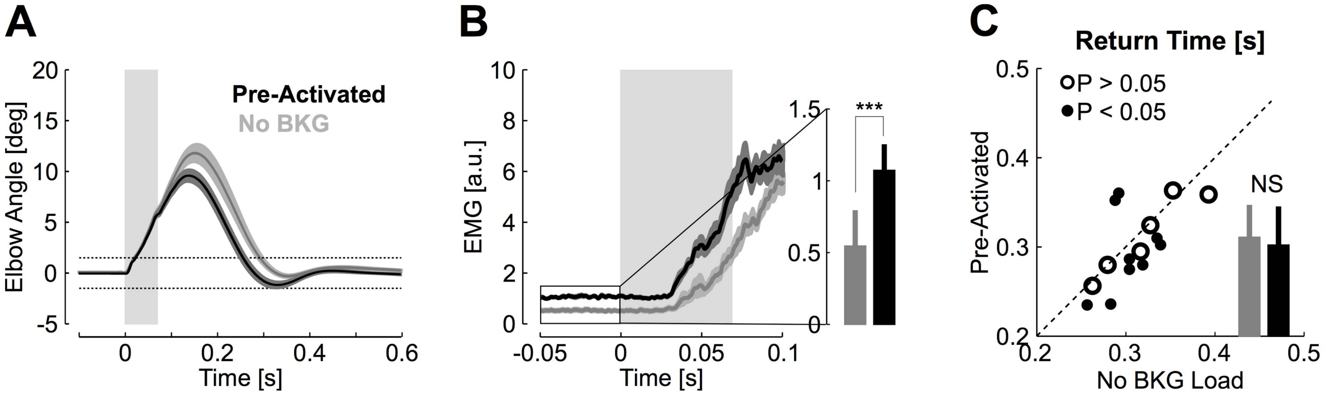

Figure 5. Effect of muscle pre-loading on return times. A: Group averages of elbow motion in the 300 ms condition, with (black) or without (gray) application of a background load. The gray rectangle illustrates the onset and offset of the square perturbation pulse. The horizontal dotted lines represent the 3 deg virtual target. Shaded areas represent 1 SEM B: Muscles grand average across subjects (mean ± SEM). The inset displays the pre-perturbation activity (−50 to 0 ms) with the same color code as in panel A (1 bar represent one standard deviation). The statistical difference across conditions is illustrated (see Results). C: Return times in the 300 ms condition without background load, plotted against the return times obtained after pre-loading the muscles. Filled dots illustrate significant differences from individual trials based on Wilcoxon ranksum tests. Open dots are displayed otherwise. Summary of group average and standard deviation is presented in the bar graph.
doi:10.1371/journal.pcbi.1003869.g005

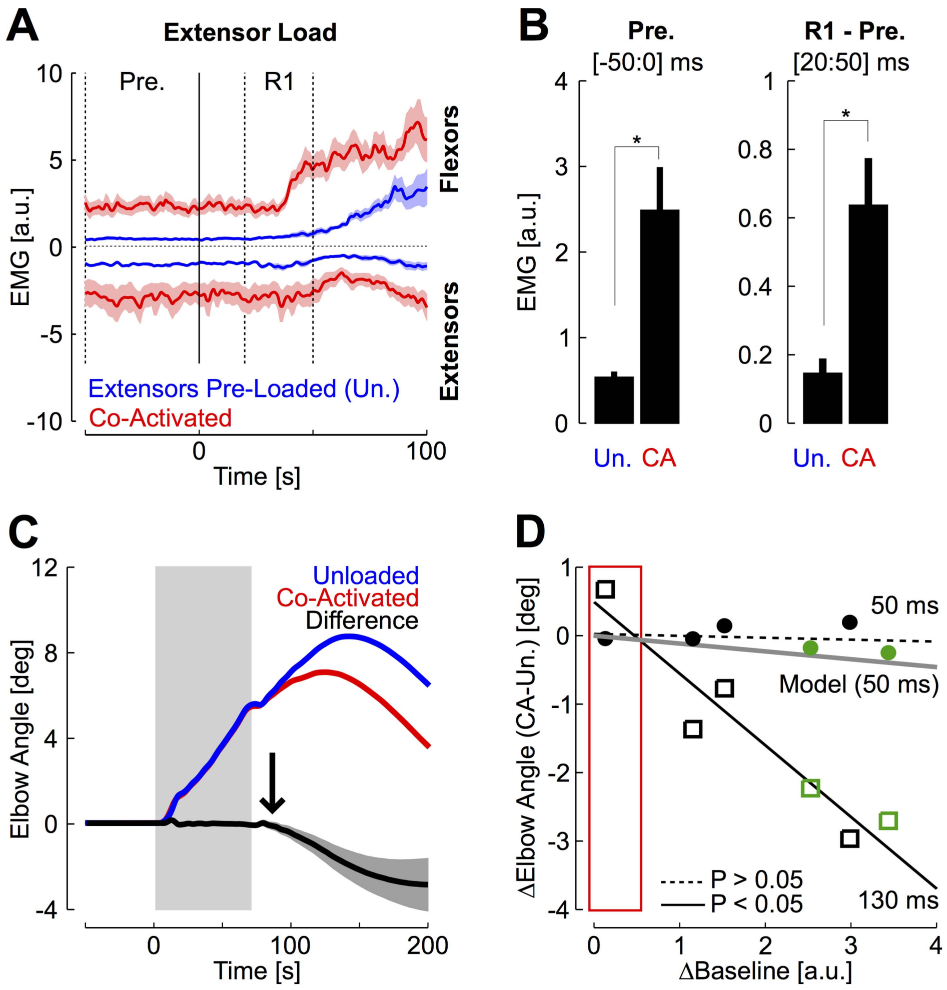

Figure 6. Effect of co-activation. A: Muscles activity averaged across groups and participants in the two conditions of muscle pre-activation following an extensor perturbation load evoking flexor response (red: co-contraction; blue: unloaded). Shaded areas are the standard error across participants. The two epochs are illustrated with vertical dashed lines (Pre.: −50 to 0 ms, R1: 20 to 50 ms) B: (Left) Changes in pre-perturbation activity across conditions when the antagonist muscle group is pre-loaded (blue, unloaded), or when muscles are co-activated (red). (Right) Difference between the muscles activity in the short-latency time window (R1: 20–50 ms) and the pre-perturbation activity. C: Elbow motion averaged across participants with similar color code as in panel 6A. The difference is plotted in black and the gray area represents the standard error of the mean across participants. The vertical rectangle illustrates the perturbation time window as in Figure 3. The vertical arrow indicates the onset of divergence estimated based on sliding t-test (~85 ms). D: Changes in elbow angle as a function of change in co-activation for individual subjects. Filled dots are the average diference in elbow angle measured at 50 ms and open squares are the average difference in joint angle measured at 130 ms. The red rectangle illustrates the range of spontaneous changes in co-activation from the main experiment. The two participants illustrated with green symbols are those for whom activation-dependent changes in joint angles occurred prior to 20 ms (see Methods).
doi:10.1371/journal.pcbi.1003869.g006

maximum average elbow displacement <5 deg, and therefore potentially exploited short-range stiffness of muscles. Observe that the associated increase in baseline is substantially greater than the spontaneous increase observed in the main experiment (the full range across participant from the main experiment is illustrated with the red frame). Group data indicated that the onset of divergence across conditions was found at ~85 ms (Figure 6B, vertical arrow), which may result from the recruitment of short-latency feedback.

To summarize, the main experiment shows that participants were able to generate very fast corrective movements with minimal use of co-contraction and without inducing systematic oscillations in the response profile. The control experiment shows that changes in co-contraction observed in the main experiment were too small to induce significant changes in perturbation-related motion.

Parameter Validation

In order to develop control models, we must first estimate parameters that best capture the visco-elastic properties of the limbs. First, it is clear that the values associated with high, short-range stiffness cannot be considered to reflect the intrinsic joint impedance for perturbations in our Main Experiment, as the perturbation-related motion was substantially greater than 5 degrees (range from Main Experiment was 8 to 20 degrees).

Estimates provided in the literature based on hand forces following perturbations typically depend on reflexes [5,7,8,12–15,28], and as a consequence do not represent the intrinsic impedance of the joint. For example, muscle impedance values commonly used in the literature ($K = 16$ Nm/rad and $G = 2.4$ Nms/rad, [29]) predict ~5 degrees of maximum joint displacement, even without considering any contribution from

neural feedback. This displacement is significantly lower than the experimentally observed joint displacement in the 600 ms condition (14.5±3.7 degrees), and even lower than observed for the 300 ms in our Main Experiment (11.6±3.2 deg, t-test between participants' individual means and theoretical maximum displacement, $t_{(14)} = 5.79$, $P<0.001$).

In light of these limitations, we used force-length and force-velocity curves to estimate the intrinsic impedance of the joints. Based on the literature, we estimated muscle stiffness (K) to be 1.61 Nm/rad and muscle viscosity (G) to be 0.14 Nms/rad (see Methods for details about the derivation). One can identify if these values are reasonable by using the data from our Main Experiment, as described below.

The following analysis estimates the set of plausible muscle stiffness and viscosity terms based on comparisons between perturbation-related motion and simulations of a passive single joint with intrinsic visco-elastic properties varied across simulations (see Methods). First, it is clear that estimates of muscle impedance cannot generate less elbow displacement than we observed in our 600 ms condition, because motion in this condition still includes some contribution of participants' neural feedback (Figure 7A). We used the measured joint displacement at 150 ms from our human subjects in the moderate temporal condition (θ_{600}) and computed the set of values of K and G that predict a displacement of the elbow joint at 150 ms equal to θ_{600}. The boundary between the values of K and G predicting a joint displacement $\theta(t)$ at 150 ms greater or lesser than θ_{600} is an upper bound on the intrinsic joint impedance. This boundary delineates the regions C and B in the parameter space represented in Figure 7 C (gray lines). Note that the commonly used values in the literature for muscle impedance lie outside of the range displayed in the diagram ($K = 16$ Nm/rad and $G = 2.4$ Nms/rad [29]).

Second, a closer estimate of a reasonable set of muscle stiffness and viscocity terms is obtained by comparing changes in the patterns of elbow motion with changes in perturbation-related EMG between the two time constraints. In the following analysis, perturbation-evoked changes in EMG are used to quantify the effect of the feedback response on the joint kinematics and extrapolate the theoretical motion of the passive joint due to muscles intrinsic properties only. We related changes in joint angle across timing conditions (Figure 7A, δA) to the corresponding changes in muscle response (Figure 7A, δR). The ratio $\delta A/\delta R$ was used to extrapolate the joint displacement at 150 ms corresponding to the intrinsic impedance (Figure 7A and B, θ_I). We used the measured joint displacement at 150 ms from our human subjects in the moderate temporal condition (θ_{600}) and the estimated joint displacement corresponding to the intrinsic properties (θ_I, open dot in Fig. 7B) to determine set of acceptable values for K and G based on simulations (thick dashed line in Fig. 7C). We then calculated the set of K and G values predicting a joint displacement at 150 ms equal to θ_I, which represents the best estimates for participants involved in the main experiment. The corresponding set represents the boundary between the regions A and B of the parameter space (Figure 6 C, black dashed lines). Observe that our estimates obtained independently (Equations 6, black dot) are in perfect agreement with the values of K and G that generate a joint displacement at 150 ms equal to θ_I.

As in any model, there are several free parameters that can be difficult to estimate (moment arm, activation level, PCSA, fascicle length and normalizing constant). Although we based our estimates on measured muscle properties to the best of our abilities (see Methods), it is clear that each value is subject to experimental measurement error. We calculated how errors in each parameter would impact the estimates of joint impedance by varying them up to ±25%. The worst-case relative change in K and G was between 50% and 160% of the initial values. These possible variations are reported in Figure 7 C with a gray rectangle. Such a range of uncertainty resulting from model parameter errors is still clearly confined within the regions A and B of the parameter space presented in Figure 7.

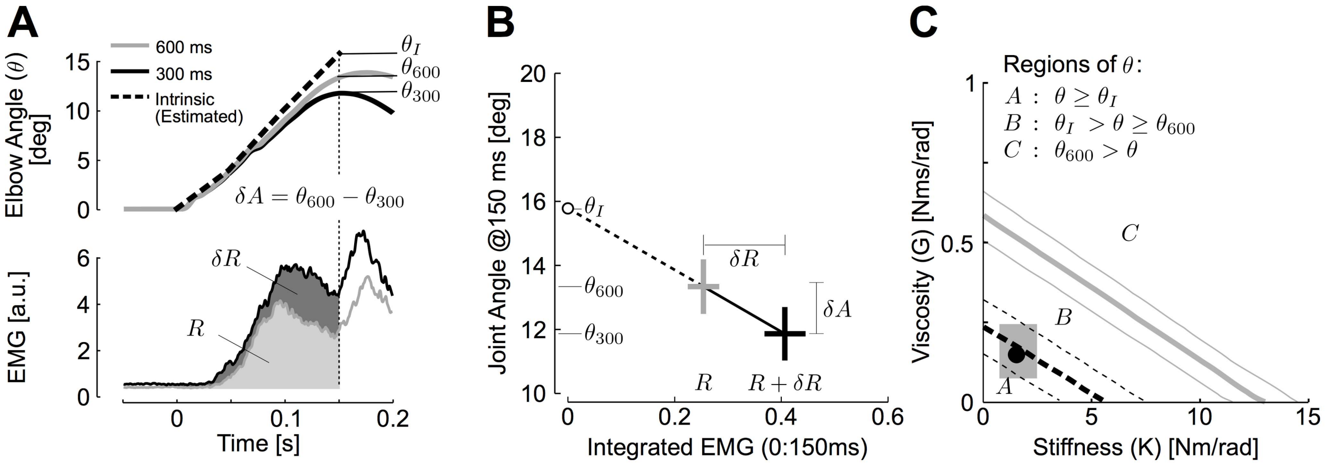

Figure 7. Validation of model parameters. A: Relationship between the integrated EMG (δR) and the corresponding change in joint angle at 150 ms (δA) across the timing conditions. The ratio $\delta A/\delta R$ was used to extrapolate the joint displacement at 150 ms resulting from the intrinsic joint impedance only (θ_I). Dashed line denotes estimated contribution generated by muscle intrinsic mechanical properties. B: Extrapolation of changes in joint angle as a function of changes in integrated muscle response used to estimate θ_I. The same extrapolation based on integrated EMG and joint angle at distinct times provided similar results. Crosses illustrate 1 SEM across participants. C: Regions of the parameter space (K and G) predicting changes in simulated joint angle at 150 ms $\geq\theta_I$ (region A), between θ_I and θ_{600} (region B) and $<\theta_{600}$. The boundary between the regions A and B is the set of values of K and G that best corresponds to our data. Upper and lower boundaries were obtained by varying the segment inertia by ±5%. The black dot represent the estimates derived from the muscle model (Equations 6). The gray rectangle shows the worst-case variation in K and G resulting from ±25% errors in the muscles model parameters (see Results).
doi:10.1371/journal.pcbi.1003869.g007

Simulations of Control Models

The purpose of the following analysis is to determine which control model can explain the ability of human subjects to perform fast and stable feedback control given low impedance and the presence of sensorimotor delays. We consider a linear model of the elbow joint coupled with a first order low-pass filter representing muscle dynamics. The equations of motion are:

$$I\ddot{\theta} = -K\theta - G\dot{\theta} + T, \quad (1)$$

$$\tau\dot{T} = u - T, \quad (2)$$

where θ is the joint angle (dots represent time derivative), I [Kgm^2] is the forearm inertia, K [Nm/rad] and G [Nms/rad] are the elastic and viscous components of the intrinsic stiffness, T is the controlled torque, τ is the time constant of the muscle and u is the control variable.

A first candidate control model is a direct mapping of the state of the system (represented by x) delayed by $\delta t = 50$ ms, compatible with long-latency delays (see Methods). This control model can be written as:

$$u = Cx(t - \delta t), \quad (3)$$

where C is a row vector of feedback gains. We analyze three candidate controllers from this class of models, each controller being represented by a row vector of feedback gains C. The first controller (C_1) minimizes the spectral abscissa, which is the rightmost eigenvalue of the closed loop control system. The spectral abscissa is directly related to the exponential decay of the joint motion towards the equilibrium following a perturbation [30], which in theory guarantees the fastest corrective movement towards the target. This controller corresponds to relatively low feedback gains (Figure 8, blue trace, $C_1 = [-2.03\ -1.07\ -0.58]$). This seems counter-intuitive since this controller should present the fastest exponential decay following perturbations. A closer look indicates that this is indeed the case, as this controller does not generate any oscillation in the corrective response, resulting in a fast decay of the angle, velocity and torque towards 0 following the perturbation. The return time obtained with this controller was 585 ms.

Although the performance of this controller is good for moderately fast return times, we are interested to generate corrective responses with return times <300 ms. Reducing the return times can only be obtained at the cost of tolerating oscillations, provided that they remain within the virtual target bounds. This is illustrated with the second controller minimizing the return times (red trace, $C_2 = [-10.81\ -2.37\ -0.98]$). The return time for this controller was 260 ms. However, it presented oscillations that exceeded those generated by human subjects (Figure 8 A, inset). It is also important to realize that this controller was quite sensitive to the presence of noise in the process, a common feature of biological motor systems [31–33]. Indeed, injecting small amounts of noise in the simulations substantially altered its performance as illustrated in Figure 8 B (dashed red histogram, average return time >350 ms). Observe that the oscillations do not vanish on average, as they are not due to noise but to the controller spectral properties. Thus, although the performance of this control candidate is good in the absence of any source of noise, we may question its relevance as a model for human behaviour on the basis that its sensitivity to process noise impedes consistent success in the fastest temporal condition.

In theory, it is possible to limit the impact of process noise by using robust control design (black trace, $C_3 = [-10.92\ -2.4\ -1.22]$), which minimizes the controller sensitivity to perturbations and uncertainties in the model parameters [30,34]. Indeed, with similar amounts of sensorimotor noise, the robust controller generates a distribution of return times that is narrower than the distribution obtained with the controller designed to minimize return times (Figure 8 B). However, improving the robustness is

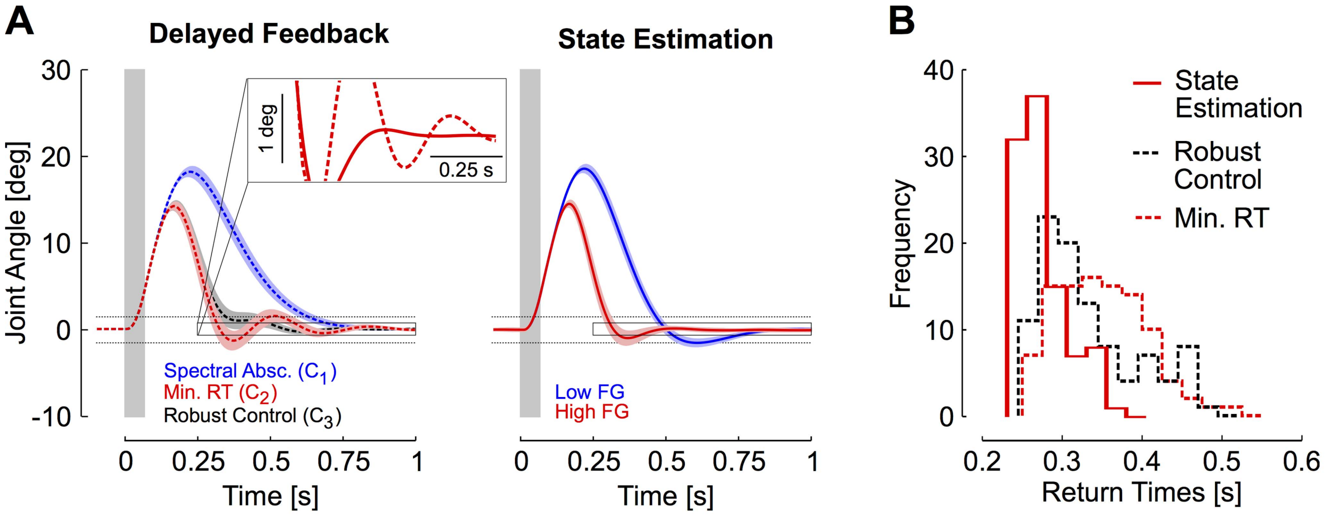

Figure 8. Simulations results. A: Simulations of feedback responses following perturbations with delay-uncompensated control (left) and feedback control based on state estimation (right). Dashed horizontal traces illustrate the virtual target and the shaded rectangle represents the perturbation duration. Feedback responses obtained with the three delay-uncompensated candidate controllers are represented: minimum spectral abscissa (blue, C_1), minimum return time (red, C_2), and maximum stability radius (robust control, black, C_3). Responses of the controller based on state estimation are displayed for two distinct cost-functions illustrating the effect of increasing the feedback gains on the overall response profiles (high feedback gains, red, or low feedback gains, blue). Areas represent a standard deviation across 100 simulation runs at each point in time. B: Distributions of return times obtained with the delayed controllers (dashed) and with state estimation (solid red).
doi:10.1371/journal.pcbi.1003869.g008

achieved to the detriment of control performance [35], as illustrated by the shift in return times towards greater values (average return time = 0.33 s, Figure 8 B). To summarize, increasing the feedback gains of delay-uncompensated controllers (as for C_2 and C_3 in comparison with C_1) reduced the return times but generated oscillations. Any attempt to increase the feedback gains to match human performance generated oscillations that exceeded the target bounds, and we were not able to find any stable delay-uncompensated controller generating consistent return times <300 ms in the presence of sensorimotor noise.

In contrast, it was much easier to reproduce participants' data with simulations based on state estimation, without any apparent limitations on the feedback gains (Figure 8 A, right and 8 B). This class of control models differs from Equation 3 in that the feedback gains are applied to an estimation of the present state of the system represented by $\hat{x}$ given by a Kalman filter:

$$u = C\hat{x}(t). \tag{4}$$

For these controllers, increasing the feedback gains simultaneously reduced the maximum joint angle as well as the target overshoot, which was the signature of participants' successful trials (Figure 2). Interestingly, the high feedback gains used with the state estimator generated unstable control when applied directly to delayed sensory input. This result highlights the advantage of state estimation to generate fast and stable feedback control, and corroborates our previous findings regarding the influence of state estimation on rapid feedback responses to perturbations [36].

Thus, the controller based on state estimation performs better than the tested delay-uncompensated feedback controllers. However, using internal models is prone to errors in the presence of model errors, causing an inherent trade-off between control performance and robustness dependent upon the accuracy of the internal model [30]. We observed the consequences of this control principle by varying the joint inertia while maintaining constant the robust controller (C_3), and the model-based controller (including feedback gains and Kalman gains). Varying the inertia by $\pm 10\%$ only induced small changes in return times obtained with the robust controller ($<10\%$ on average), in agreement with the fact that it is in theory the least sensitive to small changes in model parameters. In contrast, similar variations induced more than 20% increase in return times when using estimation-based control. The performance of the controller minimizing the return times was also quite sensitive to changes in inertia ($>30\%$ increase in return times when inertia decreases by 10%). Simulations further indicated that the robust controller started to perform better than the model-based controller if inertia changed by $\geq$ 15%. Thus, robust control is clearly a good candidate in the presence of model uncertainty when internal models of dynamics are not sufficiently accurate to ensure fast and stable control.

Finally, the simulations allow us to quantify the relative contribution of intrinsic impedance to the total torque produced against the external perturbation. In the slowest temporal condition, the peak elastic and viscous torques represent 23% and 18%, respectively, of the controlled torque generated by the feedback response. It is worth noting that the intrinsic elastic and viscous torques do not reach their peak values at the same time, nor at the peak resultant torque. The contribution of the passive torques represents 22% of the peak resultant torque. The relative contribution of the joint intrinsic stiffness is reduced in the fastest temporal condition because the feedback response limits the perturbation-related motion. In this condition, the intrinsic stiffness and viscosity represent 9.6% and 9.4% of the peak controlled torque respectively, and their combined action contributes to 10% of the peak resultant torque.

Discussion

We show that participants are able to generate very fast corrective movements following mechanical perturbations without substantial oscillations and while using only small increases in pre-perturbation activity. Based on anthropometric data, observed perturbation-related motion, muscle recordings and a full muscle model [23,24], we derived a linear model of the elbow joint with realistic feedback delays and intrinsic impedance. With these parameters, we found that state estimation is an easy and effective way to permit fast corrective movements (return times <300 ms).

Estimating the joint stiffness and viscosity was central to our analyses because it critically influences the predictions of each control model. The conventional technique for estimating joint stiffness is with servo-controlled perturbations to extract the relationship between the hand displacement and the restored force [5,7,12,13,15,28]. One shortcoming of this technique is that the restored force measured over ~200 ms includes the contribution of short-latency (>20 ms), long-latency (>50 ms) and early voluntary feedback responses (>100 ms) [3,37–39]. Thus, this approach provides estimates that include not only the intrinsic mechanical impedance of the joint, but also neural feedback. We estimated joint stiffness and viscosity from the mechanical properties of muscle. Our sensitivity analysis may not fully capture estimation errors that result from using a linear muscle model. However, some ignored non-linear features (such as plateaus in force-velocity curves and the presence of elasticity in the tendon, see Methods) would result in estimates of the muscles visco-elastic properties that are even smaller than our first-order approximations. It is clear that future studies would be useful to explore the limitations of these linear approximations to address whether the basic results presented in this paper remain valid in general.

We wish to emphasize that we do not reject the presence of peripheral joint impedance. We show that short-range impedance can contribute substantially against transient perturbations during postural control. However, short-range impedance cannot counter perturbations during movement or when abrupt perturbations induce large motor errors. Thus, what we question is the contribution of joint intrinsic impedance during movement and feedback responses to moderate-sized disturbances, given that perturbation-related motion quickly overcomes the short-range stiffness (in ~60 ms in the main experiment). Previous work also reported that the intrinsic components of muscles only provide limited contribution to force feedback in comparison with neural feedback [40]. Further, short-range impedance cannot contribute during voluntary movements, as it is only present in static conditions. These observations indicate that feed-forward regulation of intrinsic joint impedance, suggested as the first level of sensorimotor control hierarchy [9,41], may not play a substantial role during voluntary control.

This is puzzling because co-contraction is often observed as a spontaneous strategy [42–44]. We also found a significant (small) increase in baseline activity across timing conditions. However, higher levels of joint stiffness and viscosity can only be obtained by increasing baseline activation substantially (see the Control Experiment), yet nobody chose this strategy despite the failure rate. Why then, in some conditions, does the brain use co-contraction? Perhaps co-contraction is mostly beneficial to counter small disturbances by exploiting the short-range stiffness.

In order to understand the role of co-contraction, one must consider not only its contribution peripherally, but also its

contribution centrally to feedback processing. It has been shown that perturbations applied with higher background levels of muscle activity lead to larger short-latency stretch responses [45–47] (see also the Control experiment). This gain-scaling quality of the short-latency spinal reflex is likely due to the size recruitment principle of motoneurons, whereby the motor units are recruited in order of their strength [48]. Thus, by increasing the level of baseline activation, spinal feedback can recruit stronger motor units with faster contraction times, increasing the gain of spinal feedback and hastening the corrective response. Like the short-range stiffness, it is worth pointing out that spinal gain-scaling is potentially deleterious as the short-latency stretch response lacks most of the sophisticated capabilities present in long-latency responses [2]. In fact, this increase in gain is transient, as it disappears within 100 ms after the perturbation, during the long-latency time period [47].

In general, our data do not allow us to make any definitive statement regarding how neural circuits express model-based control, and it is clearly possible that the spinal cord is engaged in addition to supra-spinal and cortical contributions. However, we provide empirical evidence that participants did not strongly engage short-latency spinal responses in this task, as measured response onsets occurred at $\sim$50 ms on average. The control experiment also revealed that the spontaneous increase in co-activation observed in the main experiment was very small in comparison with the range in which the benefits of co-contraction are apparent. Thus, although the short-latency spinal stretch reflex generates a faster muscle response, it was clearly not exploited by participants. It should be noted that this strategy may have depended on the task instruction. We focused on timing constraints because return times are directly affected by the boundedness of the set of stabilizing delayed-feedback controllers, making the limitations of such controllers easier to observe. Other tasks for which stability and model-based control are less critical (such as shooting through a target) may allow other strategies such as more recruitment of intrinsic impedance and short-latency feedback.

The present results on the limited contribution of short latency spinal reflexes appears to be at odds with the classic studies by Nichols and Houk [40] that highlight spinal reflexes in decerebrate cats can compensate for changes in muscle stiffness. However, this study demonstrated that this spinal-based compensation was only possible with sufficient background muscle activity. At low levels of background muscle activity, spinal reflexes were insufficient to counter the change in muscle stiffness properties [40], consistent with the observations in the present study.

State estimation has been exploited to characterize unperturbed reaching during which the brain may rely on internal predictions from forward models and efference copy of motor commands [49–52]. Although this hypothesis is firmly established in the context of voluntary movements, evidence of estimation underlying rapid feedback responses has remained elusive. Previous studies provide indirect evidence for state estimation driving feedback control, without dissociating the rapid update based on sensory feedback about the perturbation from the prediction of the effects of the motor commands [53–56]. Recently we showed that internal priors about the perturbation profiles were engaged in the long-latency response before sufficient sensory information was collected to accurately respond to the perturbation [36]. This previous study showed that long-latency responses were not purely dependent upon sensory feedback; instead these responses were compatible with a rapid update of the estimate state of the limb using internal knowledge of the perturbation profiles. The present paper highlights the benefits of this state estimation to provide rapid feedback control following perturbations (and more generally during voluntary control).

However, our simulations also highlight potential strategies for delay-uncompensated feedback control to provide relatively fast corrective responses. In particular, robust control is beneficial to reduce the impact of errors in model parameters, but at the cost of greater return times that can impede task success. Robust control may also provide important insight on the role of Golgi tendon organs (GTO). This sensory organ provides feedback about the muscle force [57,58], but its role remains controversial. Interestingly, the robust controller has higher absolute torque feedback than the other delay-uncompensated controllers. This results from the fact that maximizing the stability radius requires that the closed-loop control system is as far as possible from the unstable bounds, and the system becomes quickly unstable with non-negative torque feedback. Thus, increasing negative torque feedback improves the system's stability margin, but also slows down corrective feedback. This theoretical feature of robust control is compatible with the regulatory action of the GTO [57]. More generally, the inherent trade-off between performance and robustness, previously reported in a bimanual task [59], is likely an important feature of online feedback control that requires further study. It is possible that biological motor systems select control solutions that achieve performance or robustness depending on the quality and reliability of body and environment's internal models of dynamics.

Our results have important implications for motor learning and adaptation given the link between feedback control and motor learning [60]. Indeed, previous studies addressing adaptive changes in movement control have predominantly focused on trial-by-trial adjustment of the descending commands [11,29,61–64]. This approach is partially supported by the fact that several modeling studies have considered very high impedance values [29,54,65–67], or have ascribed them to short-latency spinal pathways [53,54], which in both cases substantially overestimates the actual properties of the limb (see Figure 7C). Thus, simulations obtained with such models predict that online corrections for motor errors encountered while moving in a novel dynamical environment are handled by the intrinsic properties of the limbs or by high-gain short-latency reflexes. It seems important to re-evaluate this aspect of motor learning theory and re-explore the mechanisms underlying online feedback control while exposed to unknown dynamics. Robust control is again a good candidate model to capture motor strategies during early exposure to unknown dynamics, giving place to a greater reliance on internal models following the acquisition of motor skills. In theory, it is also possible to adjust internal models within a single movement with rapid reverberating loops mapping motor errors into model updates [68]. We expect that future work on motor learning will shed light on how the motor system handles model errors during online feedback control.

The rapid drop in muscle stiffness beyond a short range of motion may explain the presence of pre-synaptic inhibition of direct spinal feedback during movement [69]. There is an extensive literature highlighting that a group of GABA inhibitory interneurons in the spinal cord form synapses on the axons of sensory afferents that terminate onto motor neurons and interneurons [70]. These GABA interneurons generate presynaptic inhibition on sensory afferents during the transition from posture to movement [70,71]. Pre-synaptic inhibition effectively reduces the gain of sensory feedback at the level of the spinal cord, and it has been presumed that this is necessary to extract task-relevant information about movement [70,72]. Although plausible, it is unclear why information about self-generated motion is

irrelevant to the central nervous system, nor how presynaptic inhibition on synapses between sensory afferents and motoneurons relate to sensory processing.

We believe that pre-synaptic inhibition may also have a functional role for motor function. Indeed, limb movement also results in muscle switching from high to low impedance [73]. Thus, it is possible that pre-synaptic inhibition reflects a relative shift in the contribution of spinal feedback. During postural control, the motor system can exploit short-range impedance of muscles and relatively elevated spinal gains. However, during movement when muscle possesses low stiffness, direct spinal feedback is reduced and the central nervous system exploits to a greater extent on internal models and state estimation that is expressed in long-latency motor responses.

How these mechanisms, purely spinal and supra-spinal, interact is not straightforward. However, this question appears to be at the core of how the nervous system maintains stable interactions with the environment. In the present study, we emphasized that upper limb stability is threatened by state-dependent muscle mechanics as well as sensorimotor delays. Stability issues also arise when interacting with intrinsically unstable environments, such as when one manipulates non-rigid objects or when stepping on unsteady ground. Using a paradigm that can reproduce such situations, Lawrence and colleagues [74] recently showed that humans displayed consistent capabilities to stabilize finger or lower-limb forces against unstable springs across healthy and clinical populations. Altogether, these observations suggest that the interaction between peripheral and central mechanisms is likely a core challenge for the nervous system in most tasks. Simple hierarchical models have been suggested [9,41,75], but this view still leaves open the problem of central compensation for state-dependent properties of the lower level of the hierarchy such as the transition from high to low impedance as well as task-dependent spinal feedback. Alternatively, the brain may selectively rely on short-range control or model-based control depending on the task or on the perturbation-related motion. In this framework, it is possible that pre-synaptic inhibition regulates spinal sensorimotor gains to complement muscles biomechanics and partially compensate for changes in their properties across postural control and movement tasks.

Methods

Ethics Statement

The Queen's University Research Ethics Board approved the experimental protocol and participants gave written informed consent following standard procedures.

Experimental Procedures

A total of 16 healthy volunteers (11 males) between 19 and 33 yrs of age took part in this study. Fifteen participants performed the main experiment. Five of them also performed the control experiment. One participant was tested for the control experiment only.

Main experiment. Participants (N = 15) interacted with an adjustable robotic linkage supporting their right arm against gravity and allowing motion in the horizontal plane (KINARM, BKIN Technologies, Kingston, ON) [76,77]. Visual targets and feedback about the fingertip position (1 cm radius white circle) were projected on a virtual reality display while direct vision of the arm was blocked (Figure 2 A). The shoulder joint was physically locked and perturbations only induced motion at the elbow joint.

The time course of a trial is represented in Figure 2 C. The start target (red dot, radius = 0.6 cm) and goal target (red circle, radius = 1 cm) were presented at 90 deg of elbow angle. Participants were instructed to stabilize in the start target and compensate for the perturbations applied after a random delay uniformly distributed between 2 s and 4 s. The perturbations applied on the elbow were square pulses of 5 Nm amplitude applied for 50 ms with 10 ms build up/down (Figure 2 B). The perturbations were applied with three different levels of background load: extensors pre-excited (Figure 1 B, $L_0 = +2$ Nm), flexors pre-excited ($L_0 = -2$ Nm) or no pre-loading ($L_0 = 0$ Nm). Flexion and extension perturbations were randomly interleaved to avoid anticipation. The task was to return within an imposed time constraint and stabilize the fingertip in the goal target for 2 sec (Figure 2 C, t_{MAX}). Task success was displayed with a green goal target when participants returned and stabilized within the prescribed time limit. Otherwise, the goal target remained red, indicating an unsuccessful trial (Figure 2 C). Visual feedback of the index fingertip was always displayed.

We varied the time constraints to return to the spatial goal to compare response profiles across timing conditions with theoretical simulations. Participants first performed a block of 40 trials (20 × flexion or extension pulses) per pre-loading condition (3 blocks) with $t_{MAX} = 600$ ms, followed by a second similar series of three blocks with $t_{MAX} = 300$ ms. The blocks were separated by short pauses of a few minutes to avoid fatigue. Participants were aware from the beginning of the experiment that the second series of three blocks would test their fastest response. There was no lower time limit applied in the 600 ms condition and some participants already attempted to respond as quickly as possible.

Control experiment. This experiment aimed to test the effect of muscles co-activation on rapid feedback responses to perturbations. Participants (N = 6) performed three blocks of trials identical to those of the main experiment. One of these blocks was performed with an extensor background load (−2 Nm), another block was performed with a flexor background load (+2 Nm) and the third block was performed without any background load, but with the explicit instruction to increase muscles co-activation to make the elbow joint as rigid as possible, while being able to maintain this level of co-activation relatively constant across the 40 trials. This instruction was provided only for the block of trials in which there was no pre-loading. The temporal requirement for the control experiment was 300 ms.

Data Collection and Analysis

The elbow angle, velocity and the activity of the major muscles spanning the elbow joint were sampled at 1 kHz. Position signals were digitally low-pass filtered with a dual pass 4th order Butterwoth filter with 50 Hz cutoff frequency. The activity of elbow muscles was collected with surface electrodes (DE-2.1, Delsys, Boston, MA) attached over the muscle belly after light abrasion of the skin with alcohol. We collected the activity of bi- and mono-articular elbow flexors and extensors (brachioradialis, biceps, triceps lateralis and triceps long). Muscle recordings were digitally band-pass filtered with a dual-pass, 4th order Butterworth filter (band-pass 10–400 Hz), rectified and averaged across trials. Normalization was performed relative to the activity evoked by a 2 Nm background load when maintaining postural control at the start target (90 degrees of elbow angle). We present the ensemble-averaged activity as we found qualitatively similar behaviour across muscles.

We extracted the maximum elbow angle following the perturbation and the maximum target overshoot when returning to the goal. The maximum target overshoot was computed relative to the centre of the goal target. We also extracted the return times, defined as the time when the elbow angle returned within ±1.5 deg of the initial angle, corresponding to a virtual goal target of 3 degrees centered on the start position. Trial success was

determined offline based on the return time of each individual trial. Comparisons of parameters from individual trials across conditions were performed for each participant independently with a non-parametric Wilcoxon ranksum test. Group comparisons across conditions were based on paired t-tests performed on each participants' individual means.

The control experiment addressed the onset of divergence between perturbation-related changes in joint angles across pre-activation conditions. The onset of divergence across trials was computed for each participant based on time series of ROC areas following procedures described earlier [78]. We also addressed the onset of divergence across participants. For this analysis, a time series of paired t-tests was preferred in order to mitigate the impact of inter-participants variability. For each analysis (ROC on individual trials or running t-tests across participants), we extracted the divergence onset as the last chance-crossing time.

Derivation of Model Parameters

The estimate of joint intrinsic stiffness and viscosity is based on the static force-length/velocity curves as described in [23,24]. The normalized fascicle tension (F) is a function of the normalized muscle activation level (a), length (L) and velocity (V). The normalized tension must be multiplied by a constant proportional to the physiological cross-sectional area to estimate the total muscle force (S), and by the muscle moment arm to estimate the muscle torque (d). Thus, the resultant muscle torque is given by:

$$T = d \times S \times F(a,L,V). \tag{5}$$

The intrinsic joint impedance (Equation 1) is the ratio between changes in the joint torque and the changes in joint angle or velocity. Thus, a first approximation, the intrinsic elastic (K, [Nm/rad]) and viscous (G, [Nms/rad]) component of the muscle impedance is given by computing the derivative of the muscle torque with respect to muscle length or velocity as follows ($p_0 = [a_0,\ L_0,\ V_0]^{\mathrm{T}}$ is the parameter vector around which the derivatives were computed):

$$K = \left[\frac{\partial T}{\partial L}\right]_{p_0} \left[\frac{\partial L}{\partial \theta}\right]_{\theta_0}, \quad G = \left[\frac{\partial T}{\partial V}\right]_{p_0} \left[\frac{\partial V}{\partial \dot{\theta}}\right]_{\dot{\theta}_0}. \tag{6}$$

The numerical values used to compute K and G were either measured or taken from the literature. We used $d = 4$ cm for the moment arm [79,80]. The physiological cross-sectional areas (PCSA) and muscle fascicle lengths were measured on human muscles samples from 9 cadavers following standard techniques (see Supporting Information) [81]. We considered the sum of PCSA over muscle groups (flexors or extensors), and averaged it across groups and individuals. The average PCSA across human muscle samples was 16.38 cm^2, which must be multiplied by the maximum tension generated per square centimeter (31.8 N/cm^2, [23]), which gives $S = 520.9$ N. Muscles fascicle length was also measured for each muscle group, and averaged across samples ($L = 13.38$ cm) to calculate the relationship between changes in joint angle and changes in normalized length and velocity. We added 0.05 Nms/rad to the constant G to account for joint friction independent from muscle dynamics. To estimate the level of activation a_0, we calculated the activation needed to produce 2 Nm joint torque according to Equation 5, and used the fraction corresponding to the pre-perturbation activity measured in the 300 ms condition averaged across participants (54% of the activity evoked by 2 Nm background load). The other values used in Equation 6 were $L_0 = 1$, $V_0 = \dot{\theta}_0 = 0$ and $\theta_0 = 90$ deg. With these parameters, we obtained $K = 1.61$ Nm/rad and $G = 0.14$ Nms/rad. The short-range stiffness was obtained by estimating the slope for force-length relationship based on Rack and Westbury [22] (Figure 2 in this reference, ~7 N/mm), and scaling this number to human muscles properties. This computation gave us an elastic stiffness 9.4 times greater than the value obtained from the static force-length curve (~15 Nm/rad, see also [17]). The viscosity was scaled by the same factor to generate the simulations with short-range impedance presented in Figure 1.

We validated these parameters by comparing simulations of a passive joint following perturbation pulses. The motion was generated by considering a system corresponding to Equation 1 with $T = 0$. We varied the parameters K and G across simulations and compared the perturbation-related motion with participants' data from the main experiment. This approach allowed us to derive sets of values for K and G representing upper bounds and admissible combinations given participants' data (Figure 7).

The equations of motion and control models were defined in Equations 1–4. The system inertia was estimated based on average anthropometric data [82] and on the robot linkage mass and geometry ($I = 0.11$ Kgm^2). The time constant of the muscle model (Equation 2) is $\tau = 66$ ms, compatible with first order approximation of muscle dynamics [24]. Sensorimotor delays were measured as the time when muscle responses exceed 2 standard deviation of their baseline activity. Individual onset times were averaged across muscles and participants (Figure 9, 50.5 ms$\pm$5.5 ms, mean $\pm$ SD across participants). This value corresponds to long-latency delays typically observed in absence of muscle pre-loading [2].

One limitation of our approach is to consider a linear model of muscles visco-elastic properties, which is clearly a crude approximation given the non-linearity of muscles biomechanics [24]. Two important points can be examined: first we ignored the contribution of heterogeneous connecting tissues acting in series with contractile tissues in the quantification of the overall elastic component of muscles. Second, we ignored that the force-velocity relationship rapidly plateaus for moderate joint velocities [23]. Observe these two modeling choices yield an overestimation of the parameters K and G. Indeed, our approach effectively considers that tendons have infinite stiffness and therefore likely overestimate the actual elastic force beyond the short range. This approximation is partially justified by the fact that tendon stiffness was reported to be much greater than the stiffness of the contractile elements [83]. Similarly, considering a constant viscosity across all velocities overestimates the true (time varying) muscle viscosity resulting from the non-linear force-velocity curve.

Identification techniques are sometimes used in the literature to quantify the contribution from intrinsic and reflexive components of joint torque following a perturbation [16,17]. Although the values of stiffness can clearly vary across joints and muscles due to different properties, several estimates published with this technique seem extremely high (>100 Nm/rad), and almost certainly non physiological. One potential problem with this approach is that it is often based on fitting procedures, and the conditioning of the fit is not systematically verified. As a result the fitting procedure may by extremely sensitive to model errors and data variability. An important question for future studies is to investigate the origin of disagreement between this approach and ours.

Control Models and Simulations

We considered three candidate controllers corresponding to Equation 3, minimizing the spectral abscissa, the sensitivity to

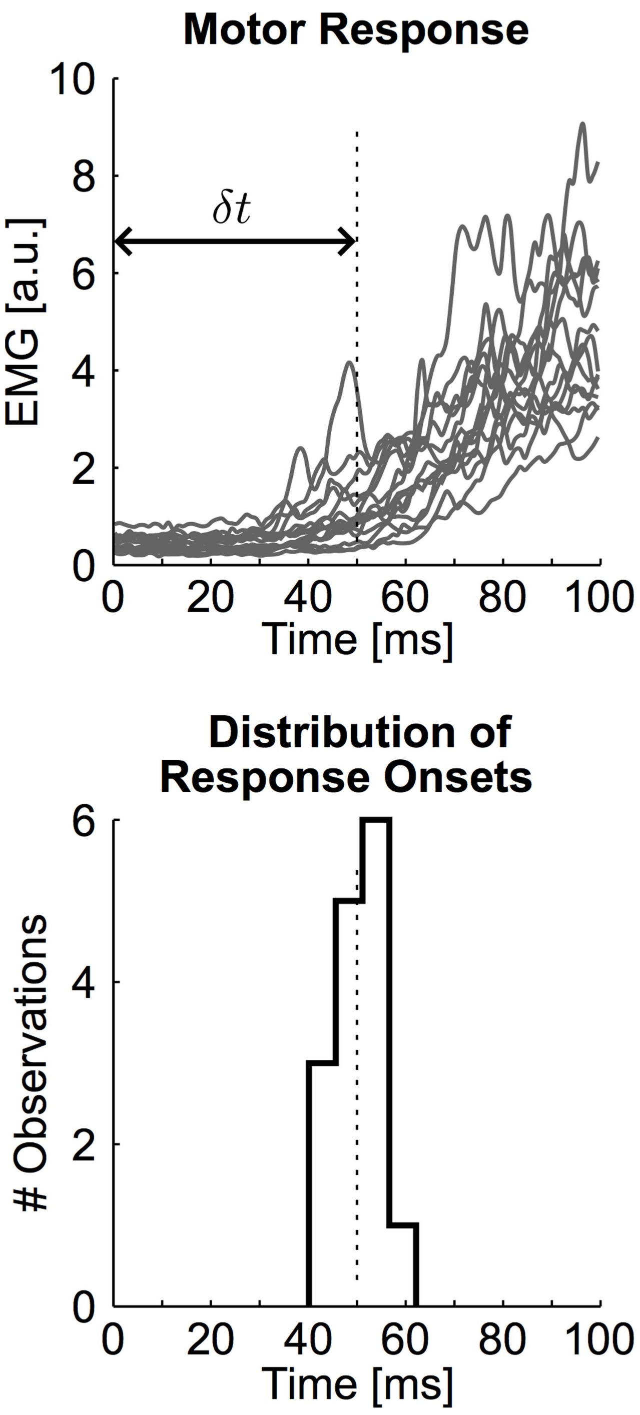

Figure 9. Feedback delay. Top: Individual muscle responses used to define the feedback delay (δt). The vertical dashed line illustrates the average response onset. Bottom: Histogram of response onsets across participants.
doi:10.1371/journal.pcbi.1003869.g009

parameters error (robust control) and the return times. The eigenvalues of the closed-loop system were computed with the freely available Matlab package DDE-BIFTOOL [84]. Each optimization procedure was based on first order evaluation of the sensitivity of the objective function (spectral abscissa, stability radius or return time) relative to the feedback gains [30].

The second class of control models corresponding to Equation 5 is based on an estimation of the state of the system [51,85,86]. The cost-function used for this model penalizes position errors ($\theta_t \neq 0$) and motor costs (u) as follows:

$$J=\sum_{t-1}^{N} w\theta_t^2+r_t u_t^2, \tag{7}$$

where N is the time horizon (>2 sec, 5 ms time steps), w and r_t ($1 \leq t \leq$N-1) are parameters adjusted to get return times <600 ms ($w=0.01,\ r_t=10^{-4},\ r_N=0$). We followed the procedures fully described earlier to derive the optimal Kalman gains and control gains (C in Equation 4), while taking the feedback delays into account [87,88]. We varied w to increase the feedback gains until the simulated trajectories matched participants' return times. Varying w to match participants' behaviour was the only fitting procedure used in this study. The noise parameters (additive and signal dependent) were identical for each model simulation and were not fitted to participants' data. We verified that the variability across simulations was lesser than participants' trial-to-trial variability, which ensures conservative conclusions. All other parameters were measured experimentally or taken from the literature.

Our model assumes that the brain receives feedback about the joint position, velocity and joint torque. However, previous work emphasizes that information about the joint acceleration is also encoded in the discharge rate of muscle spindles [89]. Observe that the differential equation describing joint dynamics (Equations 1 and 2) can be transformed into its canonical form in which the joint acceleration becomes a state variable as follows:

$$\left(1+\tau\frac{d}{dt}\right)\left(I\ddot{\theta}+K\theta+G\dot{\theta}\right)=u. \tag{8}$$

Thus, the systems considering torque or acceleration derivatives (Equations 2 or 8) are equivalent in the sense that similar control inputs generate the same motion. We verified that the predictions obtained with Equation 8 gave the same results as those obtained based on Equations 1 and 2.

Supporting Information

Table S1 List of muscle parameters. Physiological cross sectional areas (PCSA) and fascicle lengths of elbow flexor and extensor muscle groups from nine human cadavers.
(XLS)

Acknowledgments

We want to thank K. Moore, H. Bretzke and J. Peterson for technical and logistic support, and M. Omrani for motivating discussions.

Author Contributions

Conceived and designed the experiments: FC SHS. Performed the experiments: FC. Analyzed the data: FC SHS. Contributed reagents/materials/analysis tools: FC. Wrote the paper: FC SHS.

References

1. Miall RC, Weir DJ, Wolpert DM, Stein JF (1993) Is the Cerebellum a Smith Predictor? Journal of Motor Behavior 25: 203–216.
2. Scott SH (2012) The computational and neural basis of voluntary motor control and planning. Trends in cognitive sciences 16: 541–549.

3. Pruszynski JA, Scott SH (2012) Optimal feedback control and the long-latency stretch response. Experimental Brain Research 218: 341–359
4. Burdet E, Osu R, Franklin DW, Milner TE, Kawato M (2001) The central nervous system stabilizes unstable dynamics by learning optimal impedance. Nature 414: 446–449.
5. Mussa-Ivaldi FA, Hogan N, Bizzi E (1985) Neural, mechanical, and geometric factors subserving arm posture in humans. Journal of Neuroscience 5: 2732–2743.
6. Franklin DW, Osu R, Burdet E, Kawato M, Milner TE (2003) Adaptation to stable and unstable dynamics achieved by combined impedance control and inverse dynamics model. Journal of Neurophysiology 90: 3270–3282.
7. Perreault EJ, Kirsch RF, Crago PE (2001) Effects of voluntary force generation on the elastic components of endpoint stiffness. Experimental Brain Research 141: 312–323.
8. Selen LPJ, Franklin DW, Wolpert DM (2009) Impedance Control Reduces Instability That Arises from Motor Noise. Journal of Neuroscience 29: 12606–12616.
9. Loeb GE, Brown IE, Cheng EJ (1999) A hierarchical foundation for models of sensorimotor control. Experimental Brain Research 126: 1–18.
10. Hogan N (1984) Adaptive control of mechanical impedance by co-activation of antagonist muscles. Ieee Transactions on Automatic Control 29: 681–690.
11. Franklin DW, Burdet E, Tee KP, Osu R, Chew CM, et al. (2008) CNS Learns Stable, Accurate, and Efficient Movements Using a Simple Algorithm. Journal of Neuroscience 28: 11165–11173.
12. Burdet E, Osu R, Franklin DW, Yoshioka T, Milner TE, et al. (2000) A method for measuring endpoint stiffness during multi-joint arm movements. Journal of Biomechanics 33: 1705–1709.
13. Tsuji T, Morasso PG, Goto K, Ito K (1995) Human hand impedance characteristics during maintained posture. Biological Cybernetics 72: 475–485.
14. McIntyre J, MussaIvaldi FA, Bizzi E (1996) The control of stable postures in the multijoint arm. Experimental Brain Research 110: 248–264.
15. Gomi H, Osu R (1998) Task-dependent viscoelasticity of human multijoint arm and its spatial characteristics for interaction with environments. Journal of Neuroscience 18: 8965–8978.
16. Kearney RE, Stein RB, Parameswaran L (1997) Identification of intrinsic and reflex contributions to human ankle stiffness dynamics. Ieee Transactions on Biomedical Engineering 44: 493–504.
17. Mirbagheri MM, Barbeau H, Kearney RE (2000) Intrinsic and reflex contributions to human ankle stiffness: variation with activation level and position. Experimental Brain Research 135: 423–436.
18. Krutky MA, Trumbower RD, Perreault EJ (2013) Influence of environmental stability on the regulation of end-point impedance during the maintenance of arm posture. Journal of Neurophysiology 109: 1045–1054.
19. Matthews PBC (1991) The human stretch reflex and the motor cortex. Trends in Neurosciences 14: 87–91.
20. Strick PL (1978) Cerebellar involvement in volitional muscle responses to load changes. In: Desmedt, J E editor. Progress in Clinical Neurophysiology, Vol 4 Cerebral Motor Control in Man: Long Loop Mechanisms. Basel, Switzerland: S. Krager. pp 85–93.
21. Joyce GC, Rack PMH, Westbury DR (1969) Mechanical properties of cat soleus muscle during controlled lengthening and shortening movements. Journal of Physiology-London 204: 461–&.
22. Rack PMH, Westbury DR (1974) Short-range stiffness of active mammalian muscle and its effect on mechanical properties. Journal of Physiology-London 240: 331–350.
23. Scott SH, Brown IE, Loeb GE (1996) Mechanics of feline soleus.1. Effect of fascicle length and velocity on force output. Journal of Muscle Research and Cell Motility 17: 207–219.
24. Brown IE, Cheng EJ, Loeb GE (1999) Measured and modeled properties of mammalian skeletal muscle. II. The effects of stimulus frequency on force-length and force-velocity relationships. Journal of Muscle Research and Cell Motility 20: 627–643.
25. Morgan DL (1977) Separation of active and passibe components of short-range stiffness of muscle. American Journal of Physiology 232: C45–C49.
26. van Eesbeek S, de Groot JH, van der Helm FCT, de Vlugt E (2010) In vivo estimation of the short-range stiffness of cross-bridges from joint rotation. Journal of Biomechanics 43: 2539–2547.
27. Crevecoeur F, Kurtzer I, Bourke T, Scott SH (2013) Feedback responses rapidly scale with the urgency to correct for external perturbations. Journal of Neurophysiology 110: 1323–1332.
28. Franklin DW, Milner TE (2003) Adaptive control of stiffness to stabilize hand position with large loads. Experimental Brain Research 152: 211–220.
29. Shadmehr R, Mussa-Ivaldi FA (1994) Adaptive representation of dynamics during learning of a motor task. Journal of Neuroscience 14: 3208–3224.
30. Michiels W, Niculescu S-L (2007) Stability and stabilization of time-delay systems. Philadelphia: Society for Industrial and Applied Mathematics. 378 p.
31. Faisal AA, Selen LPJ, Wolpert DM (2008) Noise in the nervous system. Nature Reviews Neuroscience 9: 292–303.
32. van Beers RJ, Haggard P, Wolpert DM (2004) The role of execution noise in movement variability. Journal of Neurophysiology 91: 1050–1063.
33. Churchland MM, Afshar A, Shenoy KV (2006) A central source of movement variability. Neuron 52: 1085–1096.
34. Bhattacharyya SP, Chapellat H, Keel LH (1995) Robust control - The parametric approach.: Prentice Hall PTR.
35. Boulet B, Duan Y (2007) The fundamental tradeoff between performance and robustness - A new perspective on loop shaping. Ieee Control Systems Magazine 27: 30–44.
36. Crevecoeur F, Scott SH (2013) Priors Engaged in Long-Latency Responses to Mechanical Perturbations Suggest a Rapid Update in State Estimation. Plos Computational Biology 9(8) e1003177.
37. Lee RG, Tatton WG (1975) Motor responses to sudden limb displacements in primates with specific CNS lesions and in human patients with motor system disorders. The Canadian journal of neurological sciences Le journal canadien des sciences neurologiques 2: 285–293.
38. Crago PE, Houk JC, Hasan Z (1976) Regulatory actions of human stretch reflex. Journal of Neurophysiology 39: 925–935.
39. Nakazawa K, Yamamoto S, Yano H (1997) Short-and long-latency reflex responses during different motor tasks in elbow flexor muscles. Experimental Brain Research 116: 20–28.
40. Nichols TR, Houk JC (1976) Improvement in linearity and regulation of stiffness that results from actions of stretch reflex. Journal of Neurophysiology 39: 119–142.
41. Franklin DW, Wolpert DM (2011) Computational Mechanisms of Sensorimotor Control. Neuron 72: 425–442.
42. Gribble PL, Mullin LI, Cothros N, Mattar A (2003) Role of cocontraction in arm movement accuracy. Journal of Neurophysiology 89: 2396–2405.
43. Milner TE (2002) Adaptation to destabilizing dynamics by means of muscle cocontraction. Experimental Brain Research 143: 406–416.
44. Deserres SJ, Milner TE (1991) Wrist muscle actional paterns and stiffness associated with stable and unstable mechanical loads. Experimental Brain Research 86: 451–458.
45. Marsden CD, Merton PA, Morton HB (1976) Servo action in human thumb. Journal of Physiology-London 257: 1–44.
46. Matthews PBC (1986) Observations of the automatic compensation of reflex gain on varying the preexisting level of motor discharge in man. Journal of Physiology-London 374: 73–90.
47. Pruszynski JA, Kurtzer I, Lillicrap TP, Scott SH (2009) Temporal Evolution of "Automatic Gain-Scaling". Journal of Neurophysiology 102: 992–1003.
48. Monster AW, Chan H (1977) Isometric force production by motor units of extensor digitorum communis muscle in man. Journal of Neurophysiology 40: 1432–1443.
49. Miall RC, Wolpert DM (1996) Forward models for physiological motor control. Neural Networks 9: 1265–1279.
50. Wolpert DM, Flanagan JR (2001) Motor prediction. Current Biology 11: R729–R732.
51. Todorov E, Jordan MI (2002) Optimal feedback control as a theory of motor coordination. Nature Neuroscience 5: 1226–1235.
52. Shadmehr R, Krakauer JW (2008) A computational neuroanatomy for motor control. Experimental Brain Research 185: 359–381.
53. Bhushan N, Shadmehr R (1999) Computational nature of human adaptive control during learning of reaching movements in force fields. Biological Cybernetics 81: 39–60.
54. Wagner MJ, Smith MA (2008) Shared Internal Models for Feedforward and Feedback Control. Journal of Neuroscience 28: 10663–10673.
55. Ariff G, Donchin O, Nanayakkara T, Shadmehr R (2002) A real-time state predictor in motor control: Study of saccadic eye movements during unseen reaching movements. Journal of Neuroscience 22: 7721–7729.
56. Mehta B, Schaal S (2002) Forward models in visuomotor control. Journal of Neurophysiology 88: 942–953.
57. Houk J, Henneman E (1967) Response of Golgi Tendon Organs to active contractions of soleus muscle of cat. Journal of Neurophysiology 30: 466–&.
58. Mileusnic MP, Loeb GE (2006) Mathematical models of proprioceptors. II. Structure and function of the Golgi tendon organ. Journal of Neurophysiology 96: 1789–1802.
59. Ronsse R, Thonnard JL, Lefevre P, Sepulchre R (2008) Control of bimanual rhythmic movements: trading efficiency for robustness depending on the context. Experimental Brain Research 187: 193–205.
60. Cluff Y, Scott SH (2013) Rapid feedback responses correlate with reach adaptation and properties of novel upper limb loads. Journal of Neuroscience 33: 15903–15914.
61. Miall RC, Jackson JK (2006) Adaptation to visual feedback delays in manual tracking: evidence against the Smith Predictor model of human visually guided action. Experimental Brain Research 172: 77–84.
62. Flanagan JR, Vetter P, Johansson RS, Wolpert DM (2003) Prediction precedes control in motor learning. Current Biology 13: 146–150.
63. Krakauer JW, Ghilardi MF, Ghez C (1999) Independent learning of internal models for kinematic and dynamic control of reaching. Nature Neuroscience 2: 1026–1031.
64. Sing GC, Orozco SP, Smith MA (2013) Limb motion dictates how motor learning arises from arbitrary environmental dynamics. Journal of Neurophysiology 109: 2466–2482.
65. Scheidt RA, Ghez C (2007) Separate adaptive mechanisms for controlling trajectory and final position in reaching. Journal of Neurophysiology 98: 3600–3613.
66. Berniker M, Kording K (2008) Estimating the sources of motor errors for adaptation and generalization. Nature Neuroscience 11: 1454–1461.

67. Pinter IJ, van Soest AJ, Bobbert MF, Smeets JBJ (2012) Conclusions on motor control depend on the type of model used to represent the periphery. Biological Cybernetics 106: 441–451.
68. Fortney K, Tweed DB (2012) Computational Advantages of Reverberating Loops for Sensorimotor Learning. Neural Computation 24: 611–634.
69. Scott SH, Crevecoeur F (2014) Feedback throttled down for smooth moves. Nature 38:509–510.
70. Rudomin P (2009) In search of lost presynaptic inhibition. Experimental Brain Research 196: 139–151.
71. Seki K, Perlmutter SI, Fetz EE (2003) Sensory input to primate spinal cord is presynaptically inhibited during voluntary movement. Nature Neuroscience 6: 1309–1316.
72. Wolpert DM, Diedrichsen J, Flanagan JR (2011) Principles of sensorimotor learning. Nature Reviews Neuroscience 12: 739–751.
73. Axelson HW, Hagbarth KE (2001) Human motor control consequences of thixotropic changes in muscular short-range stiffness. Journal of Physiology-London 535: 279–288.
74. Lawrence EL, Fassola I, Werner I, Leclercq C, Valero-Cuevas FJ (2014) Quantification of dexterity as the dynamical regulation of instabilities: comparisons across gender, age, and disease. Frontiers in neurology 5: 53–53.
75. Raphael G, Tsianos GA, Loeb GE (2010) Spinal-Like Regulator Facilitates Control of a Two-Degree-of-Freedom Wrist. Journal of Neuroscience 30: 9431–9444.
76. Scott SH (1999) Apparatus for measuring and perturbing shoulder and elbow joint positions and torques during reaching. Journal of Neuroscience Methods 89: 119–127.
77. Singh K, Scott SH (2003) A motor learning strategy reflects neural circuitry for limb control. Nature Neuroscience 6: 399–403.
78. Pruszynski JA, Kurtzer I, Scott SH (2008) Rapid motor responses are appropriately tuned to the metrics of a visuospatial task. Journal of Neurophysiology 100: 224–238.
79. Murray WM, Delp SL, Buchanan TS (1995) Variation of muscle moment arms with elbow and forearm position. Journal of Biomechanics 28: 513–525.
80. Li W, Todorov E (2007) Iterative linearization methods for approximately optimal control and estimation of non-linear stochastic system. International Journal of Control 80: 1439–1453.
81. Cheng EJ, Scott SH (2000) Morphometry of Macaca mulatta forelimb. I. Shoulder and elbow muscles and segment inertial parameters. Journal of Morphology 245: 206–224.
82. Winter DA, editor (1979) Biomechanics of human movement: John Wiley and Sons.
83. Joyce GC, Rack PMH (1969) ISOTONIC LENGTHENING AND SHORTENING MOVEMENTS OF CAT SOLEUS MUSCLE. Journal of Physiology-London 204: 475–&.
84. Engelborghs K, Luzyanina T, Roose D (2002) Numerical bifurcation analysis of delay differential equations using DDE-BIFTOOL. Acm Transactions on Mathematical Software 28: 1–21.
85. Astrom KJ (1970) Introduction to stochastic control theory. New York: Academic Press.
86. Bryson AH, Ho Y-C (1975) Applied Optimal Control: Optimization, Estimation, and Control: Taylor and Francis Group.
87. Todorov E (2005) Stochastic optimal control and estimation methods adapted to the noise characteristics of the sensorimotor system. Neural Computation 17: 1084–1108.
88. Crevecoeur F, Sepulchre RJ, Thonnard JL, Lefevre P (2011) Improving the state estimation for optimal control of stochastic processes subject to multiplicative noise. Automatica 47: 595–596.
89. Dimitriou M, Edin BB (2008) Discharges in human muscle spindle afferents during a key-pressing task. Journal of Physiology-London 586: 5455–5470.
